# Bridging Digital Learning Competence and Academic Achievement: The Roles of Informal Digital Learning and Metacognitive Self-Regulation

**DOI:** 10.3390/jintelligence14020031

**Published:** 2026-02-13

**Authors:** Heeyoon Ko

**Affiliations:** Future Education Learning Support Center, National Korea Maritime & Ocean University, Busan 49112, Republic of Korea; hyko@kmou.ac.kr

**Keywords:** digital learning competence (DLC), informal digital learning engagement (IDLE), metacognitive self-regulation (MSR), academic achievement (AA)

## Abstract

The author investigates how digital learning competence (DLC) is bridged to academic achievement (AA) through informal digital learning engagement (IDLE) and how meta-cognitive self-regulation (MSR) shapes these pathways among university students. Grounded in a moderated mediation framework, this research conceptualizes DLC not as a static skill set but as a latent capacity that is channeled into academic outcomes when students autonomously engage in digital environments and regulate their cognition. Survey data were collected from 432 undergraduate students and analyzed using Partial Least Squares Structural Equation Modeling (PLS-SEM). The results show that DLC significantly predicts AA both directly and indirectly via IDLE, identifying informal digital learning engagement as a central pathway through which digital learning competence is translated into academic gains. Furthermore, MSR moderates the relationship between DLC and IDLE, such that higher levels of metacognitive self-regulation strengthen the conversion of digital learning competence into productive informal digital learning engagement. These findings support a dynamic view of digital learning competence and underscore the roles of learner autonomy and metacognitive awareness in transforming digital skills into meaningful educational outcomes. By integrating perspectives on digital literacy, self-regulated learning, and informal learning, this study offers implications for the design of digital learning ecosystems that effectively bridge students’ digital capacities with their academic achievement.

## 1. Introduction

In contemporary higher education, students’ learning routines and everyday academic practices are increasingly shaped by networked screens and digital platforms, extending beyond the boundaries of fixed classroom settings (H. Lee & Lee, 2024). Across Korea, university students now commute through subway tunnels, settle into cafés, and return to dorm rooms while streaming machine-learning lectures on YouTube, debating assignments on Discord, and curating portfolios via Instagram Reels—often before formal classes even begin. A nationwide post-COVID-19 survey reports that eighty-seven percent of undergraduates engage in self-directed digital learning outside scheduled coursework, marking a 340 percent increase over pre-pandemic levels (H. Lee & Lee, 2024). These developments blur the line between everyday life and academic work, elevating digital learning competence (DLC) as a central concern in educational reform and highlighting its potential to bridge ubiquitous digital activity with formal academic achievement (Burgos et al., 2023; Heidari et al., 2021; Zakir et al., 2022).

Across widely adopted international competency frameworks, DigComp 2.1 specifies twenty-one competences organized into five domains and eight proficiency levels, offering a common reference point for effective citizenship in data-rich societies (Audenhove et al., 2024; Carretero et al., 2017; X. Chen et al., 2023). Likewise, guidelines from the International Society for Technology in Education portray learners as autonomous problem-solvers who critically evaluate information and create original digital artefacts (Rodríguez-García et al., 2022; Stoika, 2023). Korean universities have closely aligned with these directions: under the government’s “Digital Talent One Million” initiative, 98.2 percent of institutions now embed competency-based digital curricula into degree programs (H. J. Kim et al., 2018; H. Lee & Lee, 2024; Machleid et al., 2020; Nyikes, 2018). Within this environment, DLC is increasingly framed as a strategic lever for connecting students’ digital capabilities with their long-term academic trajectories.

Empirical evidence indicates that proficiency with devices and software, on its own, does not automatically translate into higher grade-point averages. Recent structural-equation studies suggest that the influence of DLC on achievement is transmitted mainly through behavioral pathways, particularly informal digital learning engagement (IDLE) (Abdellatif et al., 2023; Hehir et al., 2021; Hult et al., 2024). IDLE captures voluntary activities—such as watching discipline-specific videos, participating in online study groups, and using instructional applications—that can enhance course learning by thirty to forty percent among Korean undergraduates (Han et al., 2022). Comparable findings in China and Indonesia show that digital literacy strengthens IDLE, which in turn elevates academic outcomes (Htay et al., 2022; Lin et al., 2005; Hou, 2024). Together, these results point to IDLE as a behavioral mechanism that may help bridge DLC and academic achievement.

In parallel with these developments, digital learning opportunities have expanded rapidly, especially in the wake of the pandemic (H. Lee & Lee, 2024). Yet much of the extant literature continues to focus on access to technology or on surface-level usage metrics (Carretero et al., 2017), leaving underexplored how university students actually mobilize digital resources in ways that meaningfully support academic achievement. Moreover, the joint operation of digital learning competence, informal learning behavior, and metacognitive self-regulation remains poorly understood, even though these elements may interact in complex and synergistic ways to link competence with performance (Le et al., 2022; Walker & Patel, 2018; Widowati et al., 2023). To address this gap, the present study advances a comprehensive moderated-mediation model that examines both the mechanisms (via IDLE) and the conditions (via metacognitive self-regulation; MSR) under which digital competence is converted into improved academic performance. In doing so, it responds to pressing educational challenges and offers empirically grounded insights for designing inclusive and personalized digital learning ecosystems (Cao et al., 2023; Rodríguez-García et al., 2022; Stoika, 2023). This emerging line of work gives rise to three guiding research questions.

RQ1: How does digital learning competence (DLC) influence academic performance among university students?

RQ2: How does informal digital learning engagement (IDLE) mediate the relationship between DLC and academic achievement?

RQ3: How does metacognitive self-regulation (MSR) shape the relationship between digital learning competence (DLC) and informal digital learning engagement (IDLE) among university students?

In terms of significance, the present study offers an integrated view of digital learning in contemporary higher education. Existing research has often examined the effects of digital competence or informal learning behaviors separately, but relatively few investigations have analyzed how these elements interact within a single conceptual framework that explicitly incorporates metacognitive regulation (X. Chen et al., 2023; Mehrvarz et al., 2021). Although prior work has demonstrated the predictive validity of DLC scales and their direct relationship with academic achievement, much less is known about how this competence is translated into effective informal learning behaviors. This study addresses that omission by positioning IDLE as a behavioral conduit through which DLC fosters academic gains and by proposing that this conduit becomes stronger among students with higher MSR. In doing so, it shifts attention from what learners know digitally to how they enact that knowledge in self-directed digital spaces, adding a crucial cognitive dimension to digital learning debates and clarifying the roles of IDLE and MSR in bridging DLC and academic achievement.

The moderated-mediation framework proposed here makes a distinct theoretical contribution: it illuminates not only whether, but also how, digital competence promotes academic success through IDLE under varying levels of metacognitive self-regulation (X. Chen et al., 2023; Mehrvarz et al., 2021). The focus on Korean university students—who experience intense academic pressure in an entrance-exam-driven system—provides contextually grounded findings with both local importance and wider global relevance. As digital learning ecosystems grow more complex, the results of this study offer timely guidance for policymakers and educators seeking to design inclusive, effective, and personalized digital learning interventions (Benita et al., 2021; Sari et al., 2022; Z. Yang, 2022).

Within Self-Regulated Learning theory, the benefits of digital learning are viewed as critically dependent on MSR (Susantini et al., 2021). Learners who display strong MSR set specific goals, monitor their progress, and adjust their strategies during online work, leveraging digital tools strategically rather than succumbing to distraction (H. Zhao, 2024). When MSR is weak, by contrast, even high DLC may deteriorate into inefficient browsing or procrastination, diminishing the instructional payoff of IDLE. Early interventions that employ metacognitive e-modules and AI-supported scaffolds have been shown to increase planning and monitoring scores by up to twenty-four percent and to enhance content comprehension in online reading tasks (Ng et al., 2024).

In the Korean context, however, a distinctive “digital paradox” persists. Schooling that is heavily oriented toward entrance examinations has left many first-year students with limited experience in autonomous strategy selection and reflective goal setting. Survey findings show substantial heterogeneity in DLC across cohorts and majors, suggesting that uniform, one-size-fits-all training may widen rather than narrow proficiency gaps (H. Lee & Lee, 2024). Embedding MSR workshops within DLC courses may therefore help equalize opportunities, particularly for students encountering advanced analytics platforms or maker-space technologies for the first time (Chorosova et al., 2022; Dias-Trindade et al., 2021).

Taken together, this study tackles the three research questions outlined above to model the dynamic interplay among DLC, IDLE, MSR, and academic performance in Korean higher education. By applying a moderated-mediation structure, it extends earlier work that examined only direct associations or single-mediator effects (H. J. Kim et al., 2018; Redmond et al., 2018). Ultimately, clarifying these mechanisms carries substantial policy implications. Universities must move beyond a narrow emphasis on hardware and software provision and implement dual-track interventions that cultivate metacognitive habits alongside technical skills. Such an approach holds promise for reducing learning disparities, advancing national digital-talent agendas, and redefining academic excellence in an era when the classroom is as mobile and networked as the students who inhabit it.

## 2. Literature Review

### 2.1. Unpacking the Role of Digital Learning Competence in Academic Achievement

Digital learning competence (DLC) encompasses a multifaceted set of knowledge, skills, attitudes, and strategies that enable learners to operate effectively and autonomously within digital environments (Carretero et al., 2017). The DigComp 2.1 framework, developed by Carretero et al. (2017), conceptualizes DLC across five interrelated domains: information and data literacy, communication and collaboration, digital content creation, safety and security, and problem-solving (Carretero et al., 2017). These domains reflect a comprehensive understanding of digital literacy that extends beyond technical proficiency to include evaluative thinking, ethical engagement, and adaptability in technology-mediated academic contexts. In alignment with this view, the ISTE Student Standards highlight the role of digital tools in promoting active, creative, and collaborative learning (Crompton & Burke, 2024).

Recent empirical research has validated measurement instruments that capture the multidimensional nature of digital competence, with studies demonstrating strong psychometric properties across diverse cultural and educational contexts (Morales et al., 2024; Weli et al., 2024). Furthermore, the Students’ Digital Competence Scale (SDiCoS) developed by Tzafilkou et al. (2022) identified six core components: Search, Find, Access (SFA); Develop, Apply, Modify (DAM); Communicate, Collaborate, Share (CCS); Store, Manage, Delete (SMD); Evaluate (EV); and Protect (PR), providing a comprehensive framework for assessing digital competence in higher education settings (Tzafilkou et al., 2022).

A growing body of empirical research confirms that DLC contributes meaningfully to academic achievement through multiple pathways. Proficiency in information and data literacy allows students to locate and evaluate educational resources more effectively, thereby enhancing understanding and reducing unnecessary cognitive effort (X. Chen et al., 2023). Recent meta-analytic evidence suggests a medium positive correlation between digital literacy and academic achievement, with effect sizes varying across different educational contexts and measurement approaches (H. J. Kim et al., 2018). Skills related to communication and collaboration facilitate productive peer interaction and timely feedback, which deepen students’ engagement with course content (Mehrvarz et al., 2021; Ng et al., 2024). Digital content creation supports the organization and presentation of ideas in diverse formats, enabling learners to perform more effectively in both formative and summative evaluations (Qurratu’ain et al., 2024; Zakir et al., 2022).

Furthermore, competencies in digital safety help reduce online distractions and protect against information security threats, while strong problem-solving abilities enable students to overcome technical challenges that might otherwise interfere with their academic progress (Romero et al., 2020). Recent longitudinal studies have demonstrated that students who develop agency through digital tool mastery are better prepared for future academic and professional challenges (H. Zhao, 2024). The protective effects of digital competence have become particularly evident during crisis periods, with research showing that students with higher DLC levels experienced less academic disruption and maintained better psychological well-being during remote learning transitions (Patil et al., 2021).

Empirical findings from various contexts consistently show that students with stronger digital learning competence tend to achieve better academic outcomes, even when controlling for prior academic background or individual characteristics. In this study, we adopt the measurement scale developed by Song et al. (2025), which conceptualizes digital learning competence through five subdimensions: information and data literacy, communication and collaboration, digital content creation, safety, and problem solving (Song et al., 2025). These components reflect the essential digital capabilities that enable students to navigate academic tasks in technology-rich environments. Song et al. (2025) found that digital learning evaluation competence significantly predicted undergraduate academic achievement, with the effect remaining robust after controlling for demographic variables and institutional factors (Song et al., 2025).

Mediational analyses reveal that the relationship between DLC and academic achievement often operates through intermediate processes. Zakir et al. (2022) identified that digital informal learning engagement and self-efficacy partially mediated the relationship between digital literacy and academic performance, suggesting that digital competence enhances learning outcomes by promoting autonomous learning behaviors and confidence in digital environments (Zakir et al., 2022). Similarly, X. Chen et al. (2023) demonstrated that learning adaptation and online self-regulated learning formed a sequential mediation pathway linking digital literacy to academic achievement (X. Chen et al., 2023).

Recent advances in measurement have enabled more precise assessment of the DLC-achievement relationship. The development of psychometrically validated instruments such as the Digital Competence Scale for University Students (DC-US) and the Basic Digital Competences 2.0 (COBADI^®^) questionnaire has facilitated large-scale investigations across multiple institutions and countries (Morales et al., 2024; Tzafilkou et al., 2022; Wang et al., 2021). These studies consistently report moderate to strong correlations between comprehensive DLC measures and various academic performance indicators, including grade-point averages, course completion rates, and perceived learning gains.

Information literacy, as a core component of DLC, has received particular attention in higher education research. Studies investigating the impact of information literacy on academic performance have consistently found positive associations, with students demonstrating higher information literacy skills showing improved performance in coursework, examinations, and overall academic outcomes (Widowati et al., 2023; Yousefianzadeh et al., 2020). Large-scale multi-institutional studies have revealed that information literacy activities are positively and significantly correlated with student engagement and students’ perceived gains, providing robust evidence for the academic value of these competencies (Ngozi, 2024; Widowati et al., 2023). Building on this theoretical foundation and empirical support, the following hypothesis is proposed:

**H_1_.**  *University students’ digital learning competence positively influences their academic achievement.*

### 2.2. Exploring the Connection Between DLC and Informal Digital Learning Participation

Informal digital learning engagement (IDLE) refers to students’ voluntary engagement in digital activities outside formal curricula to advance their academic development. Such activities include watching discipline-specific videos on YouTube, participating in online learning communities, or using mobile learning applications (He & Zhu, 2017; H. Lee & Lee, 2024). Unlike structured instruction guided by syllabi or instructors, IDLE is driven by learners’ autonomy and self-regulation and typically occurs beyond scheduled class hours. As higher education embraces blended and online modalities, IDLE has emerged as a key indicator of active academic involvement (Bamoallem & Altarteer, 2021; H. J. Kim et al., 2018; Suryaratri et al., 2022).

A growing body of empirical research demonstrates that DLC strongly predicts students’ levels of IDLE. In their pioneering study, He and Zhu (2017) found that Chinese undergraduates with higher DLC were significantly more likely to engage in subject-related video viewing, online study-group discussions, and self-assessment tool use (He & Zhu, 2017). Mehrvarz et al. (2021) extended these findings to Iranian university students, showing that advanced digital skills not only increased the frequency of IDLE but also amplified its academic benefits (Mehrvarz et al., 2021). Similarly, DLC enhances learners’ adaptability and self-directed behaviors in remote and flexible learning contexts, a trend accentuated by the rapid expansion of online education during the COVID-19 pandemic (X. Chen et al., 2023; F. Chen, 2025).

The relationship between DLC and IDLE can be theoretically explained through the integration of digital literacy theory and Self-Determination Theory (SDT). Stronger digital competence fosters greater confidence and intrinsic motivation in the process of exploring, evaluating, and applying information using digital tools, which, in turn, promotes autonomous engagement in supplementary learning activities beyond the classroom (Tınmaz et al., 2022; Anthonysamy, 2022). This heightened self-efficacy facilitates independent learning and supports sustained engagement by fulfilling key psychological needs—autonomy, competence, and relatedness—as proposed in SDT (Damayanty & Sofyan, 2023; Ririen & Heriasman, 2021).

Additionally, the Technology Acceptance Model (TAM) helps clarify how students’ perceptions of usefulness and ease of use influence their willingness to adopt and utilize digital tools for informal learning (Basu et al., 2022). When digital technologies are perceived as beneficial and user-friendly, learners are more likely to incorporate them into their daily academic routines (Bader et al., 2021). This tendency becomes even more apparent in contexts involving emerging technologies such as AI-based tools, where intuitive and valuable features promote deeper engagement in informal learning tasks (Gar & Idris, 2021).

The indirect effects of DLC on academic achievement through IDLE have also been empirically validated using structural equation modeling. Digital literacy has been shown to positively influence academic performance by increasing informal learning engagement, with significant indirect effects observed on outcomes such as GPA (Sutarni et al., 2021; Nurhopipah et al., 2021). Similar patterns have been reported in cross-cultural studies, reinforcing the generalizability of this mediational pathway across educational contexts (Dinh & Yen, 2024). Taken together, these findings indicate that digital competence extends beyond technical functionality, encompassing the cognitive and motivational capacities that support sustained, self-directed learning in diverse digital environments.

**H_2_.**  *Digital learning competence positively influences informal digital learning engagement among university students.*

### 2.3. IDLE and Academic Achievement

IDLE is increasingly regarded not simply as a complementary element to formal instruction but as a key factor accounting for qualitative variations in academic achievement. Engaging in personalized, self-directed digital learning activities enhances learners’ long-term academic engagement and supports the development of self-regulated learning strategies, both of which are directly linked to improved academic outcomes (Heidari et al., 2021; Li et al., 2020). Repeated and intentional use of digital tools for learning facilitates deeper understanding of subject matter, greater internalization of academic concepts, and more effective preparation for assessments and tasks (Geng, 2020; Awdziej et al., 2023).

By helping students revisit unclear concepts and bridge knowledge gaps outside the boundaries of formal education, IDLE serves as a strategic and compensatory learning practice. When learners identify weak areas and actively seek out digital resources such as instructional videos or online discussion forums to reinforce understanding, they demonstrate goal-oriented academic behavior rather than passive consumption (Ortega-Arranz et al., 2019). These practices contribute not only to measurable learning improvements but also to motivational and emotional outcomes, including enhanced conceptual clarity, reduced academic stress, and greater effectiveness in study routines.

Sustained participation in IDLE has also been associated with increased academic self-efficacy, perseverance, and a stronger orientation toward lifelong learning. Learners who independently curate and structure their digital learning experiences often report higher levels of satisfaction and academic flow. Interactive and repetitive formats—such as short instructional videos paired with quizzes, explanatory forum posts, or AI-assisted content summarization—tend to amplify learning due to their engaging design and instant feedback mechanisms (Jo & Hwang, 2024; Toma & Berge, 2024; Martin et al., 2020).

The influence of both the frequency and quality of IDLE on academic achievement has been demonstrated in higher education settings. Even after accounting for learners’ prior academic performance and socio-demographic background, IDLE continues to explain meaningful differences in students’ academic success. These findings underscore its independent contribution as a powerful determinant of academic performance. Based on this theoretical and empirical basis, the following hypothesis is proposed:

**H_3_.**  *University students’ Informal digital learning engagement positively influences their academic achievement.*

### 2.4. The Mediating Role of Informal Digital Learning Engagement

While digital learning competence (DLC) may directly influence academic achievement, numerous empirical studies suggest that its effect is often strengthened through an indirect pathway mediated by IDLE. It has been repeatedly demonstrated that merely possessing digital skills does not automatically translate into better academic outcomes; instead, educational value is realized when such competence is actively transformed into meaningful learning behaviors (Heidari et al., 2021; Salas-Pilco et al., 2022). In this regard, DLC provides the foundational capability for learners to explore and utilize digital resources, while IDLE acts as a behavioral bridge that translates this capacity into tangible academic gains.

The mediating role of informal digital learning was initially proposed in studies highlighting that students with higher levels of digital competence tend to engage more frequently in online academic activities, which are positively associated with learning outcomes (He & Zhu, 2017; Younas et al., 2022). This proposition was later validated that the influence of DLC on academic achievement can be explained, at least in part, by increased IDLE (H. J. Kim et al., 2018; Mehrvarz et al., 2021). In several cases, the direct impact of DLC became nonsignificant once IDLE was included in the model, indicating the presence of partial or even full mediation depending on the educational context. Drawing upon these theoretical and empirical grounds, the following hypothesis is proposed:

**H_4_.**  *Informal digital learning engagement mediates the relationship between digital learning competence and academic achievement among university students.*

### 2.5. The Moderating Role of Metacognitive Self-Regulation (MSR)

Metacognitive self-regulation (MSR) denotes learners’ capacity to set clear learning goals, monitor comprehension and progress, and adapt strategies or effort when necessary (Mustopa et al., 2020; Schraw et al., 2006). In digitally rich learning environments—where abundant resources and constant interruptions coexist—the strategic orchestration of one’s study behaviors critically determines whether DLC translates into effective IDLE. High-MSR students skillfully select and sequence digital tools to address precise learning objectives, curtail unfocused browsing, and orchestrate goal-oriented review or self-testing routines, thus maximizing the yield of their DLC (Sari et al., 2022; Setiawan et al., 2020). In contrast, learners with weaker MSR may command sophisticated digital skills yet squander them on passive content consumption or aimless exploration, undermining potential academic benefits (Broadbent & Poon, 2015).

Empirical interventions reinforce MSR’s catalytic role in digital contexts. Integrating metacognitive training modules and AI-driven monitoring tools into online reading exercises significantly enhanced students’ strategic use of elaboration and self-questioning techniques, leading to marked gains in task performance and engagement quality (Ortega-Arranz et al., 2019). These findings echo broader evidence that self-regulated learning strategies and metacognitive scaffolds amplify the impact of digital competencies on learning behaviors (Follmer & Sperling, 2016; Yetik, 2017; ElSayad, 2024).

In essence, two students with equivalent digital competencies will differ in their informal learning engagement if one exhibits superior metacognitive self-regulation, because that learner will more effectively channel digital skills into autonomous, goal-directed study behaviors (Bahari et al., 2020; Sartina et al., 2022). Accordingly, we hypothesize that MSR moderates the DLC→IDLE pathway such that the effect of DLC on IDLE is amplified when students demonstrate higher metacognitive self-regulation. This moderated mediation framework sets the stage for testing whether MSR enhances the translation of digital skills into proactive informal learning engagement.

**H_5_.**  *Metacognitive self-regulation moderates the relationship between digital learning competence and informal digital learning engagement among university students.*

## 3. Methodology

### 3.1. Measures

A structured questionnaire was utilized to measure college students’ DLC, IDLE, AA, and MSR. The instruments were developed based on scales with established validity and reliability from previous research, with some items modified or integrated to suit the specific purpose and target population of this study. All items were rated on a 5-point Likert scale ranging from 1 (Strongly disagree) to 5 (Strongly agree).

Digital learning competence (DLC): DLC was assessed using a 24-item scale adapted from Song et al. (2025), with the wording of some items modified to fit the context of South Korean students. The scale comprises five sub-dimensions: digital learning awareness (6 items), digital learning technical competence (6 items), digital learning engagement behavior (3 items), digital learning self-management (4 items), and digital learning evaluation competence (5 items) (Song et al., 2025). In this study, DLC was conceptualized and analyzed as a second-order construct, with these five sub-dimensions serving as its first-order factors. This scale captures students’ readiness and ability to use digital tools strategically for learning processes, from technical operation to self-management and evaluation. Sample items include, “I can skillfully use application software related to my major for learning and research,” and “I often actively participate in discussions on social media platforms such as YouTube, Instagram, Facebook, and Tik Tok”.

Informal digital learning engagement (IDLE): A 5-item scale adapted from the study by J. S. Lee and Drajati (2019) was used to measure IDLE (J. S. Lee & Drajati, 2019). While the original scale focused on “Informal digital learning for English,” the items in this study were modified to relate to the students’ general majors and adapted to the South Korean context. The items assess the frequency of students’ self-initiated learning activities in digital environments outside formal coursework. Sample items include, “I learn about my major by frequently watching or listening to digital media, such as YouTube and podcasts,” and “I learn about my major by reading digital materials, such as websites and blogs”.

Academic achievement (AA): AA was measured with a 5-item scale adapted from the Subjective Academic Achievement Scale by (Stadler et al., 2021). The items were modified to align with the context of the present study. This measure reflects students’ self-evaluations of their recent academic performance relative to their peers and personal expectations. Sample items include, “I believe my academic achievement is high compared to my peers,” and “I have achieved good results on recent exams and assignments”.

Metacognitive self-regulation (MSR): This construct was measured using a 9-item scale adapted (ElSayad, 2024). This is an established, previously validated scale that captures how learners plan, monitor, and adjust their learning strategies and attention during study. The scale was modified for the context of this study; for instance, the concept of “reading” was broadened to “learning,” and “skimming” was specified as “understanding the overall structure or core objectives.” Sample items include, “I ask myself questions to check if I am properly focused on my learning when studying for my major courses,” and “Before studying new material for my major in depth, I first try to understand its overall structure or core objectives”.

Marker Variable (Social Desirability): To address potential common method bias, a 3-item social desirability scale was included. This scale was utilized as a marker variable to detect and control for common method variance.

### 3.2. Data Collection

The target population for this study was defined as undergraduate students enrolled in higher education institutions (2-year and 4-year colleges) in South Korea. Data was collected through an online survey administered by one of South Korea’s largest panel survey firms, utilizing their extensive student panel. To ensure the suitability of the sample, the study employed a non-probability sampling strategy that combined purposive and quota sampling methods. Purposive sampling was used at the initial stage, with screening questions included at the beginning of the survey to select only currently enrolled students who voluntarily agreed to participate.

To enhance the diversity and representativeness of the sample, a quota sampling method was implemented based on four criteria: university location, academic system, academic year, and major field. The specific quotas were as follows: 80 participants from each of the five national regions (Seoul metropolitan area, Gangwon/Jeju, Chungcheong, Gyeongsang, and Jeolla); 200 participants each from 2- or 3-year colleges and 4- to 6-year universities; 100 participants from each academic year (1st, 2nd, 3rd, and 4th year or higher); and 50 participants from each of the eight major fields (Humanities, Social Sciences, Arts and Physical Education, Natural Sciences, Engineering, Marine Agriculture/Fishery, Medicine/Pharmacy, and Interdisciplinary Studies).

This study was granted an exemption from review by the Institutional Review Board (IRB) at Korea Maritime and Ocean University (Approval No: KMOU-IRB 2025-05), as it was classified as low-risk research. All procedures adhered to strict ethical guidelines. Prior to the survey, participants were presented with an information sheet detailing the study’s academic purpose, the voluntary nature of their participation, and the estimated time commitment. Participants were assured of confidentiality and anonymity, with a statement that the collected data would be used for academic purposes only and permanently deleted after three years. It was also clarified that the survey contained no personally identifiable information. Participants were informed of their right to withdraw at any time without penalty; however, they were also notified that due to the anonymous nature of the data collection, it would be technically impossible to identify and delete individual responses after submission. Consent was obtained when participants voluntarily agreed to proceed with the survey. As a token of appreciation for their time and contribution, participants who completed the survey received compensation equivalent to approximately $1.50 USD.

Initially, 400 participants completed the survey. After data cleaning, responses were excluded if they were incomplete, deemed unreliable (e.g., patterned responses), or completed in under three minutes, which was considered insufficient time given the estimated 5–10 min duration of the survey. This process resulted in a final valid sample of 375 participants for the analysis.

### 3.3. Data Analysis

Data analysis was conducted using IBM SPSS Statistics ver. 26.0 and AMOS ver. 26.0 software. This study employed the two-step approach recommended (Anderson & Gerbing, 1988). In the first step, a confirmatory factor analysis (CFA) was performed on the measurement model to ensure its validity and reliability. To assess construct validity, both convergent and discriminant validity were examined. Discriminant validity was verified using the Fornell–Larcker criterion and the Heterotrait–Monotrait ratio of correlations (HTMT) (Fornell & Larcker, 1981). The reliability of the constructs was confirmed by calculating Cronbach’s alpha coefficients.

In the second step, after validating the measurement model, the structural model was analyzed to test the proposed hypotheses. Hypotheses 1 through 3 were tested by examining the statistical significance of the path coefficients within the structural model. To test Hypothesis 4, which pertains to the mediating effect of informal digital learning engagement, the bootstrapping method proposed by Preacher and Hayes (2008) was utilized (Preacher & Hayes, 2008). Finally, Hypothesis 5, which posits a moderating effect of metacognitive self-regulation, was tested through a multi-group analysis. For this analysis, participants were divided into high and low metacognitive self-regulation groups based on the mean score. A chi-square difference test was then conducted to determine if there were statistically significant differences in the path coefficients between the two groups.

## 4. Results

### 4.1. Sample Profile

The demographic and academic characteristics of the 375 participants are summarized in Table 1. The sample consisted of 182 male (48.5%) and 193 female (51.5%) students. The mean age of the participants was 21.67 years (SD = 3.14). In terms of age distribution, the largest group was 18–20 years old (45.6%), followed by 21–23 years old (29.6%), 24–26 years old (16.0%), 27–29 years old (5.3%), and 30 or older (3.5%). The distribution of class level was relatively even: freshmen comprised 25.6% (*n* = 96), sophomores 25.1% (*n* = 94), juniors 24.0% (*n* = 90), and seniors or higher 25.3% (*n* = 95) of the sample. The sample was also balanced geographically, with participants from the Capital Region (20.0%), Gwandong or Jeju (20.5%), Hoseo (19.5%), Yeongnam (20.3%), and Honam (19.7%). Regarding institutional type, 188 students (50.1%) were enrolled in 2- or 3-year colleges, and 187 (49.9%) were in 4-year or more universities. The sample included 101 students (26.9%) from public institutions and 274 (73.1%) from private institutions. Finally, participants were distributed evenly across eight academic disciplines: Humanities (12.8%), Social Sciences (12.3%), Arts and Kinesiology (12.5%), Natural Sciences (12.3%), Engineering (12.5%), Marine Agriculture, Fishery (12.8%), Medicine and Pharmacy (12.5%), and Interdisciplinary Studies (12.3%).

### 4.2. Measurement Model

#### 4.2.1. Normality Test

The normality of the data was assessed by examining the skewness and kurtosis of each measured variable. The assumption of normality is generally considered tenable if the absolute value for skewness is below 3 and for kurtosis is below 10. The results of the normality test are presented in the provided table (Kline, 2023). The skewness values ranged from −0.627 to 0.255, and the kurtosis values ranged from −0.519 to 0.442. Since all absolute values for skewness and kurtosis were well within the recommended thresholds, the data was deemed sufficiently normal. Therefore, the subsequent analyses were conducted using the maximum likelihood estimation (MLE) method, which assumes multivariate normality.

#### 4.2.2. Common Method Variance

Since all data were collected from a single source at one point in time, several procedural and statistical remedies were employed to address the potential issue of common method variance (CMV). Procedurally, several steps were taken during the survey design to minimize this bias. These included: (1) randomizing the presentation order of the items, (2) creating psychological separation by presenting the measures for each construct in distinct sections with their own instructions, (3) emphasizing the anonymity and confidentiality of all responses, and (4) ensuring that all items were written in neutral and clear language to avoid leading participants toward a specific answer.

In addition to these procedural remedies, a statistical diagnosis was conducted using a marker variable approach, as recommended by Podsakoff (Podsakoff et al., 2003). A three-item social desirability scale was included in the questionnaire to serve as the marker variable. The results of this analysis are presented in the accompanying table. The analysis shows that the differences between the factor loadings of the original measurement model and the model incorporating the marker variable were negligible, with a maximum difference of only 0.011. Furthermore, the variance in the items explained by the social desirability marker variable was generally low, with squared loadings from the marker ranging from 0.000 to 0.161. The stability of the original factor loadings, combined with the procedural remedies implemented, suggests that common method variance is not a significant concern in this study.

#### 4.2.3. Construct Validity and Reliability

The measurement model was refined by removing two items that exhibited low factor loadings. The removed items were one from the ‘digital learning self-management’ sub-scale (“I can categorize and manage various digital learning tools”) and one from the ‘metacognitive self-regulation’ scale (“If there is something I find difficult to understand during a major class, I make sure to review and clarify it afterwards”). The overall fit of the final measurement model was assessed and demonstrated an acceptable fit to the data: χ^2^ = 1790.922, df = 768 (*p* < .001), χ^2^/df = 2.332, CFI = 0.921, SRMR = 0.054, and RMSEA = 0.060 (90% CI = 0.056–0.063). These indices meet the commonly accepted criteria for model fit, indicating that the measurement model is appropriate (Henseler et al., 2014).

The results for the reliability and validity of the constructs are detailed in Table 2. The reliability and convergent validity of the constructs were then evaluated. As shown in Table 2, all constructs exhibited excellent internal consistency. Cronbach’s alpha (α) values ranged from 0.902 to 0.959, and composite reliability (CR) values ranged from 0.904 to 0.931. Since all values surpassed the recommended threshold of 0.70, the reliability of all measures was confirmed.

Convergent validity was supported by the standardized factor loadings and the average variance extracted (AVE). All standardized factor loadings were statistically significant (*p* < .001) and ranged from 0.722 to 0.935, which is well above the commonly recommended minimum of 0.50. Furthermore, the AVE for each construct, which ranged from 0.620 to 0.805, exceeded the suggested threshold of 0.50 (Fornell & Larcker, 1981). These results provide strong evidence for the convergent validity of the measurement model.

Finally, discriminant validity was assessed to ensure that the constructs in the model were empirically distinct from one another. This was evaluated using two methods: the Fornell–Larcker criterion and the HTMT. According to the Fornell–Larcker criterion, the square root of the AVE for each construct should be greater than its correlation with any other construct. As presented in Table 3, the square root of the AVE for each construct (the diagonal values, ranging from 0.792 to 0.829) was greater than all corresponding inter-construct correlation coefficients. Additionally, all HTMT ratio values were below the recommended threshold of 0.85 (Henseler et al., 2014), with the highest value being 0.744. The results from both tests confirm that all constructs in this study possess adequate discriminant validity.

### 4.3. Structural Model

#### 4.3.1. Path Coefficient Test

After confirming the validity and reliability of the measurement model, the structural model was analyzed to test the proposed hypotheses. The structural model demonstrated an acceptable fit to the data: χ^2^ = 1269.313, df = 487 (*p* < .001), χ^2^/df = 2.606, CFI = 0.925, SRMR = 0.052, and RMSEA = 0.066 (90% CI = 0.061–0.070).

The results of the path analysis for the direct effects are presented in Table 4. All three proposed hypotheses were supported. First, Hypothesis 1, which posited that DLC would positively affect AA, was supported (β = 0.270, t = 4.234, *p* < .001). Second, Hypothesis 2 proposed a positive relationship between DLC and IDLE, which was also supported (β = 0.694, t = 10.847, *p* < .001). Finally, Hypothesis 3, which suggested that IDLE would positively influence AA, was supported as well (β = 0.545, t = 8.076, *p* < .001).

#### 4.3.2. Mediation Test

To test Hypothesis 4, which proposed that IDLE would mediate the relationship between DLC and AA, a bootstrapping analysis was conducted. Following the recommendations of Preacher and Hayes (2008), 2000 bootstrap resamples with 95% bias-corrected confidence intervals were used to test the significance of the indirect effect (Preacher & Hayes, 2008).

The results, presented in Table 5, revealed a significant indirect effect of DLC on AA through IDLE (Indirect Effect = 0.459, SE = 0.066, *p* = .001). The 95% confidence interval for the indirect effect did not contain zero [0.355, 0.631], confirming that the mediating effect of IDLE is statistically significant. The analysis also showed that the direct effect of DLC on AA remained significant after accounting for the mediator (Direct Effect = 0.327, SE = 0.075, *p* = .001). These findings indicate that IDLE partially mediates the relationship between DLC and AA, thus supporting Hypothesis 4.

#### 4.3.3. Moderation Test

To test Hypothesis 5, which posited that MSR moderates the relationship between DLC and IDLE, a multi-group analysis was conducted. Participants were divided into a low MSR group (n = 190) and a high MSR group (n = 185) based on the mean score of the MSR construct. An invariance test was then performed to compare the path coefficient from DLC to IDLE between the two groups. The results of the multi-group analysis are presented in Table 6. First, metric invariance was established, as the model constraining the factor loadings to be equal across groups (Model-λ) did not show a statistically significant chi-square difference when compared to the baseline model (Model-0) (Δχ^2^ = 30.761, Δdf = 30, *p* = .251). This confirmed that the measurement scales were perceived similarly by both groups, allowing for a meaningful comparison of the structural paths.

Next, the structural path of interest from DLC to IDLE was constrained to be equal across the low and high MSR groups (Model-β). Comparing this fully constrained model to the metric invariance model (Model-λ) yielded a statistically significant chi-square difference (Δχ^2^ = 7.025, Δdf = 1, *p* = .008). This result indicates that the strength of the relationship between DLC and IDLE is not equal and varies significantly across the two groups. An examination of the path coefficients revealed that the effect of DLC on IDLE was significantly stronger for the high MSR group (β = 0.810, *p* < .001) than for the low MSR group (β = 0.436, *p* < .001). These findings confirm the significant moderating effect of MSR, thus supporting Hypothesis 5.

## 5. Conclusions

### 5.1. Discussion of Findings

This study provided empirical validation for all five hypotheses and clarified how DLC is bridged to AA through both a direct route and the differentiated roles of IDLE and MSR. Hypothesis 1 showed that DLC directly contributes to AA, reinforcing emerging perspectives that conceptualize digital skills not merely as technical utilities but as foundational enablers of academic success in digitally saturated learning environments (Carretero et al., 2017; Mehrvarz et al., 2021). In contrast to earlier studies reporting mixed results regarding the academic value of digital skills (Malecki & Elliot, 2002), the present findings underscore that when DLC is strategically applied—particularly in learner-directed contexts—it forms a direct pathway to improved academic outcomes.

Hypotheses 2 and 3 further demonstrated that DLC significantly predicts IDLE, which in turn exerts a strong positive effect on AA. Within the overarching theme of bridging DLC and AA, these results assign IDLE a central behavioral role: it operates as a key conduit through which digital skills are translated into measurable academic gains. Rather than reinforcing a strict boundary between formal and informal learning, the findings suggest that informal digital engagement constitutes a cognitively rich, self-regulated learning space with tangible academic consequences (Montilla et al., 2023; Burgos et al., 2023). In line with this, Hypothesis 4 confirmed a partial mediation effect of IDLE between DLC and AA, indicating that the behavioral channel is indispensable for realizing the full academic value of digital competencies. This extends behavioral mediation models of digital learning by highlighting learner agency in informal contexts as a core mechanism in the DLC–AA linkage (Heidari et al., 2021; Acuña-Torres et al., 2024).

Finally, Hypothesis 5 established that MSR moderates the pathway from DLC to IDLE, revealing a critical metacognitive role in the bridging process. Students with high MSR are more capable of converting digital skills into productive learning behaviors, thereby strengthening the indirect bridge from DLC to AA through IDLE. This supports the view that metacognitive awareness is not merely a complementary trait but a boundary condition that amplifies the educational return on digital investments (Wang et al., 2021; Jenkins & Demaray, 2015). Together, the moderating role of MSR and the mediating role of IDLE empirically substantiate the paper’s central claim: DLC contributes to academic achievement both directly and by flowing through behavioral and metacognitive pathways that work in tandem to bridge digital competence and academic success.

### 5.2. Theoretical Contributions

This research introduces a paradigm shift by reconceptualizing DLC as a dynamic construct whose impact on AA emerges through its interaction with behavioral engagement and cognitive regulation, rather than as a static, standalone skill set. Instead of treating digital competence as a fixed end-state, the study frames it as latent potential that is activated when learner autonomy and metacognitive control are present (Mustopa et al., 2020; Summiya & Hussain, 2024). In theoretical terms, this reframing extends the DigComp 2.1 framework by moving from “what students know digitally” to “how they strategically apply digital competencies” in authentic learning contexts, thereby specifying the basis on which DLC can form a bridge to academic achievement. This reconceptualization aligns with contemporary perspectives that emphasize the dynamic and contextual nature of digital competence, acknowledging that technical proficiency alone cannot ensure educational effectiveness without the mediating and moderating influence of strategic learning behaviors and metacognitive awareness (J. Zhao et al., 2022; Sukarno & Widdah, 2020).

The study’s most significant theoretical contribution lies in its integration of previously separate lines of inquiry into an explicit moderated-mediation framework that bridges digital literacy, informal learning, and metacognitive regulation within a single model. This integrated perspective departs from the fragmented approach of earlier work, in which these constructs were typically examined in isolation or through simple bivariate links (Summiya & Hussain, 2024; Balgabayeva et al., 2024). By empirically operationalizing digital autonomy as a measurable construct and demonstrating its role in shaping academic achievement, the research extends Self-Determination Theory into informal learning ecosystems, providing robust support for the idea that autonomy, competence, and relatedness function meaningfully in technology-mediated environments (Ryan & Deci, 2000). Within this framework, metacognitive self-regulation emerges as a critical boundary condition that strengthens the indirect pathways through which DLC is channeled into AA, thereby specifying how the bridge from digital competence to achievement is contingent on both behavioral and metacognitive processes.

Furthermore, this research challenges institutional over-reliance on formalized digital instruction by repositioning informal digital learning as a legitimate and powerful academic process that plays a central role in the DLC–AA linkage. The findings show that IDLE is not merely a supplementary activity; it operates as a critical mediating mechanism that transforms individual digital competence into measurable academic outcomes (K. T. Kim, 2019; Marić & Sakač, 2018). Conceptually, this repositions IDLE as a behavioral conduit that helps bridge DLC and academic achievement, rather than as peripheral “extra” learning. This theoretical move has important implications for how institutions conceptualize learning boundaries, suggesting that the traditional formal–informal dichotomy is less adequate in digital contexts where learning extends across settings and modalities. The study thus offers a theoretical basis for understanding how students’ autonomous engagement with digital resources outside formal instruction can substantially enhance academic performance, broadening the definition of what counts as meaningful academic behavior in the digital era.

The theoretical framework advanced in this study also contributes to ongoing discussions of metacognitive regulation as a boundary condition in educational technology research. By showing that students with higher levels of metacognitive self-regulation are significantly more capable of converting digital competence into productive learning engagement, the findings provide empirical support for models that foreground cognitive self-management in technology-enhanced learning environments (Sukarno & Widdah, 2020; Sholihah et al., 2023). This extends theoretical understanding by demonstrating that metacognitive regulation functions not as an isolated factor but as a multiplicative amplifier that shapes the strength of the behavioral bridge from DLC to AA. The moderated-mediation model reveals that the relationship between digital competence and academic achievement is not purely direct; rather, it depends on both the quality of informal learning engagement and the robustness of students’ metacognitive self-regulatory capabilities. In doing so, it offers a more nuanced theoretical account of how DLC is translated into academic success through the combined roles of informal digital learning and metacognitive self-regulation, in line with the central theme of bridging digital learning competence and academic achievement.

### 5.3. Practical Implications

The practical implications of this study extend far beyond traditional educational boundaries, calling for institutional, pedagogical, and policy reforms that explicitly recognize how DLC can be bridged to AA through the combined roles of IDLE and MSR. At the institutional level, the findings urge a shift from tool-centric digital training toward learner-centric ecosystem design that intentionally supports the behavioral and metacognitive pathways identified in this study. Universities need to move from viewing digital competence as a discrete skillset to understanding it as a dynamic capacity that requires environments which activate DLC through opportunities for informal engagement and metacognitive regulation, thereby enabling its full academic impact (Oliynyk et al., 2024; Kapasheva et al., 2024).

Modern educational institutions, therefore, should design digital learning environments that not only provide access to tools but also scaffold the bridging processes from DLC to AA. This includes integrating adaptive scaffolding mechanisms, AI-powered feedback systems, and reflective journaling platforms directly into learning management systems so that students are guided toward strategic, self-regulated learning behaviors without disrupting their natural learning flow. AI-integrated learning management systems can offer personalized learning paths, adaptive assessments, and real-time progress monitoring that enhance both IDLE and metacognitive awareness, thereby strengthening the indirect pathways from DLC to achievement (Ha et al., 2024). These technological innovations must be coupled with purposeful pedagogical design that embeds metacognitive prompts throughout coursework, encouraging students to reflect on their learning, monitor comprehension, and recalibrate strategies based on performance feedback and self-assessment.

Educators, in turn, require professional development that goes beyond technical tool mastery to a deeper understanding of how to foster cognitive self-management and metacognitive awareness as core components of the DLC–AA bridge. Faculty training programs should emphasize the cultivation of student autonomy through learning experiences that balance structured guidance with space for self-directed exploration, helping students develop the regulatory skills needed to sustain productive IDLE in complex digital landscapes (Srivastava & Dangwal, 2021; Tondeur et al., 2023). Curricular innovation should systematically integrate informal learning opportunities within formal course structures, creating hybrid models that recognize and leverage students’ autonomous digital engagement while also providing scaffolding and assessment mechanisms that ensure academic rigor and clearly link IDLE to course-level learning outcomes.

At the policy level, national digital talent initiatives must be reconceptualized as dual-pillar strategies that simultaneously prioritize technical skill acquisition and cognitive self-regulatory development. The findings suggest that infrastructure-focused approaches are insufficient if they do not also cultivate the metacognitive and behavioral capacities that allow DLC to translate into academic success. Korea’s “Digital Talent One Million” initiative and comparable international programs would benefit from integrated designs that combine digital literacy training with systematic development of metacognitive skills, ensuring that public investments in digital education foster sustainable learning capabilities rather than temporary technical proficiencies (T.-C. Yang, 2023). Policy frameworks should extend evaluation criteria beyond access metrics to include indicators of learner autonomy, self-regulation, and long-term learning transfer, thereby focusing on the full bridge from DLC to AA.

Furthermore, policy initiatives must acknowledge informal digital learning as a legitimate and essential component of the DLC–AA linkage. Educational policies should promote micro-credentialing and competency-based assessment systems capable of capturing and validating outcomes achieved through IDLE, creating pathways for learners to receive recognition for knowledge and skills gained via autonomous digital engagement (Ryhtä et al., 2021; Cabaron, 2023). Such frameworks would help bridge formal and informal learning contexts by aligning institutional recognition with the behavioral mechanisms that this study identifies as central to translating DLC into academic achievement, while maintaining quality standards and portability of credentials across educational and professional settings.

The integration of these practical implications requires sustained commitment to systemic change that treats digital learning competence as a multifaceted construct realized through coordinated development of technical skills, autonomous informal learning behaviors, and metacognitive regulatory capabilities. Successful implementation will demand that institutions move beyond fragmented initiatives toward comprehensive ecosystem thinking that aligns technological infrastructure, pedagogical practice, faculty development, student support services, and policy frameworks around a common goal: creating environments in which DLC, IDLE, and MSR work together to bridge digital competence and academic achievement, enabling students to grow into self-regulated, digitally competent learners capable of thriving in increasingly complex professional and civic contexts.

### 5.4. Limitations and Future Directions

Despite the robustness of this study, several limitations should be acknowledged. First, reliance on self-reported measures may introduce bias, although validated tools and procedural safeguards were employed. Future research should incorporate objective indicators—such as digital trace analytics, LMS logs, and academic transcripts—to validate findings and reduce common method bias (J. Zhao et al., 2022). Second, the cross-sectional design restricts causal inference. Longitudinal and experimental studies are necessary to examine how DLC, IDLE, and MSR evolve over time and influence one another dynamically (Montilla et al., 2023). Multi-wave panel designs tracking these variables across semesters or academic years would provide deeper insights into developmental trajectories. Additionally, the sample was not evenly distributed by university type (public vs. private), which may have introduced institutional-context bias. Future studies should consider stratified sampling or multi-group comparisons to examine whether the proposed relationships differ across university types. Moreover, disciplinary context may condition how digital learning competence translates into IDLE and academic achievement, given that pedagogical practices and digital tool use often vary by subject area. Future research should test subject-area differences (e.g., via measurement invariance and multi-group analysis) to assess the robustness of the model across disciplines. Third, the cultural context of the study, rooted in South Korea, invites both caution and opportunity. While the findings reflect the digital maturity and educational rigor of Korean higher education, they require validation across diverse cultural and technological contexts. Comparative studies that explore how cultural dimensions, resource access, and educational values moderate these mechanisms would enhance generalizability (Ivanec & Defar, 2023). Finally, integrating affective and motivational constructs such as digital anxiety, intrinsic motivation, and emotional regulation may offer a richer understanding of the psychological dynamics at play. Future intervention studies should explore integrated programs that simultaneously build digital competence, stimulate informal engagement, and cultivate metacognitive regulation—preferably through randomized controlled trials (Awan et al., 2021).

## Figures and Tables

**Table 1:** Respondents’ demographics and academic characteristics.

Categories	Frequency	Proportion
Gender		
Male	182	48.5
Female	193	51.5
Age		
18–20 years old	171	45.6
21–23 years old	111	29.6
24–26 years old	60	16.0
27–29 years old	20	5.3
30 or older	13	3.5
Class level		
Freshman	96	25.6
Sophomore	94	25.1
Junior	90	24.0
Senior or higher	95	25.3
Geographical region		
Capital Region	75	20.0
Gwandong or Jeju	77	20.5
Hoseo	73	19.5
Yeongnam	76	20.3
Honam	74	19.7
Institutional type by program length		
2-year (or 3-year) college	188	50.1
4-year (or more) university	187	49.9
Funding type		
Public	101	26.9
Private	274	73.1
Academic discipline		
Humanities	48	12.8
Social Sciences	46	12.3
Arts and Kinesiology	47	12.5
Natural Sciences	46	12.3
Engineering	47	12.5
Marine Agriculture, Fishery	48	12.8
Medicine and Pharmacy	47	12.5
Interdisciplinary Studies	46	12.3

**Table 2:** Measurement items and loadings.

Latent and Observed Variables	Loadings
Digital learning competency (α = 0.959; AVE = 0.656; CR = 0.904)	
Digital learning awareness	0.766
Digital learning technical competence	0.906
Digital learning engagement behavior	0.722
Digital learning self-management	0.705
Digital learning evaluation competence	0.925
Digital learning awareness (α = 0.924; AVE = 0.673; CR = 0.925)	
AW1: I can distinguish digital learning from other learning methods.	0.848
AW2: I am willing to use mobile phones, computers, tablets, and other digital devices for learning.	0.820
AW3: When encountering learning problems, I am willing to use digital methods to solve them.	0.769
AW4: I am willing to actively explore new functions of digital learning platforms and tools.	0.864
AW5: I believe digital learning can improve my learning efficiency.	0.753
AW6: I believe digital learning can enhance my learning outcomes.	0.861
Digital learning technical competence (α = 0.907; AVE = 0.620; CR = 0.907)	
TS1: When encountering problems, I can quickly determine which digital tool to use.	0.745
TS2: I can obtain needed learning resources through the Internet.	0.751
TS3: I can adapt to the operational requirements of different digital learning platforms and tools.	0.819
TS4: I can skillfully use application software related to my major for learning and research.	0.803
TS5: I can use multimedia tools and software, such as image editors and video editors, to create and edit digital learning content.	0.805
TS6: I can independently solve technical problems that may arise in digital learning, including software failures and network issues.	0.799
Digital learning engagement behavior (α = 0.924; AVE = 0.805; CR = 0.925)	
EB1: I often actively participate in discussions on social media platforms such as YouTube, Instagram, Facebook, and Tik Tok.	0.848
EB2: I often communicate and collaborate with teachers through social media tools.	0.935
EB3: I often communicate and collaborate with classmates through social media tools.	0.907
Digital learning self-management (α = 0.923; AVE = 0.802; CR = 0.924)	
SM1: I can categorize and manage learning resources obtained from the Internet.	0.864
SM3: I can establish clear learning plans and goals, and control learning progress.	0.933
SM4: I can allocate learning time reasonably to prevent procrastination and time waste.	0.888
Digital learning evaluation competence (α = 0.922; AVE = 0.707; CR = 0.924)	
EC1: I can evaluate the credibility and effectiveness of obtained digital learning resources.	0.832
EC2: I can evaluate the advantages and disadvantages of digital learning platforms and tools I use.	0.817
EC3: I can evaluate the advantages and disadvantages of digital learning environments.	0.885
EC4: I can evaluate my own learning outcomes.	0.816
EC5: I evaluate my learning outcomes through course grades.	0.853
Informal digital learning engagement (α = 0.915; AVE = 0.688; CR = 0.917)	
IL1: I learn about my major by frequently watching or listening to digital media, such as YouTube and podcasts.	0.805
IL2: I learn by communicating about topics related to my major in online communities (e.g., forums, social media).	0.811
IL3: I informally learn about my major using online games or apps.	0.841
IL4: I learn about my major by reading digital materials, such as websites and blogs.	0.824
IL5: I learn by independently searching for and using digital materials related to my major.	0.864
Academic achievement (α = 0.902; AVE = 0.654; CR = 0.904)	
AA1: I believe my academic achievement is high compared to my peers.	0.789
AA2: I consistently strive to achieve my academic goals.	0.829
AA3: I have achieved good results on recent exams and assignments.	0.761
AA4: I tend to receive academic recognition from my professors and peers.	0.844
AA5: I am satisfied with my grades at school.	0.818
Metacognitive self-regulation (α = 0.930; AVE = 0.627; CR = 0.931)	
MS1: I ask myself questions to check if I am properly focused on my learning when studying for my major courses.	0.828
MS2: Before studying new material for my major in depth, I first try to understand its overall structure or core objectives.	0.840
MS3: If I get confused about something while studying for my major, I go back and review it until I fully understand it.	0.764
MS4: If I find the material for my major difficult to understand, I change my learning approach.	0.825
MS5: I ask myself questions to make sure I understand the material I’ve learned in my major courses.	0.804
MS6: Rather than passively accepting the material in my major courses, I try to think deeply about a topic to understand its core principles.	0.773
MS7: When studying for my major courses, I try to identify which concepts I don’t understand well.	0.757
MS8: When I preview or review for my major courses, I set learning goals to plan the direction of my studies.	0.736

Model fit: χ^2^_(768)_ = 1790.922; χ^2^/df = 2.332; CFI = 0.921; SRMR = 0.054; RMSEA = 0.060 (CI = 0.056–0.063). Notes: Items SM2 and MS9 were eliminated due to low factor loadings. All factor loadings are significant (*p* < 0.001).

**Table 3:** Descriptive statistics, inter-construct correlations and discriminant validity.

	Mean	SD	DLC	IDLE	AA	MSR
Digital learning competency (DLC)	3.521	0.758	0.810	0.689	0.647	0.510
Informal digital learning engagement (IDLE)	3.392	0.865	0.694	0.829	0.744	0.559
Academic achievement (AA)	3.568	0.746	0.649	0.734	0.809	0.634
Metacognitive self-regulation (MSR)	3.206	0.780	0.516	0.555	0.624	0.792

Notes: The diagonal shaded cells represent the square root of the AVE. The values in the lower triangular matrix are the bivariate correlation coefficients, while the values in the upper triangular are the HTMT ratios. All correlation coefficients are statistically significant (*p* < 0.001).

**Table 4:** Results of path analysis for structural model.

Paths	Coefficients		Standard Errors	t-Values	Decisions
	Unstandardized	Standardized			
H1: DLC ⇒ AA	0.327	0.270	0.077	4.234 ***	Supported
H2: DLC ⇒ IDLE	1.036	0.694	0.096	10.847 ***	Supported
H3: IDLE ⇒ AA	0.443	0.545	0.055	8.076 ***	Supported

Model fit: χ^2^_(487)_ = 1269.313; χ^2^/df = 2.606; CFI = 0.925; SRMR = 0.052; RMSEA = 0.066 (CI = 0.061–0.070). Notes: DLC = digital learning competency; AA = academic achievement; IDLE = informal digital learning engagement. *** *p* < 0.001.

**Table 5:** Results of mediation test for informal digital learning engagement.

Types of Effect	Effect Sizes	95% Confidence Intervals		Standard Errors	*p*-Values
		Lower Bounds	Upper Bounds		
Total Effect	0.786	0.649	0.955	0.078	0.001
Direct Effect	0.327	0.186	0.487	0.075	0.001
Indirect Effect	0.459	0.355	0.631	0.066	0.001

Note: Results are based on 2000 bootstrap resamples with the 95% bias-corrected confidence intervals.

**Table 6:** Results of moderation test for metacognitive self-regulation.

Path	Low MSR (n = 190)		High MSR (n = 185)			Differences	Evaluation
	Std. β	t-Value	Std. β		t-Value		
DLC ⇒ IDLE	0.436	4.767 ***	0.810		8.551 ***	0.373	Supported
Models	χ^2^ (DF)		Model Comparison		RMR	CFI	RMSEA
			Δχ^2^ (ΔDF)	*p*-Value			
Model-0	1955.846 (974) ***				0.059	0.897	0.052
Model-λ	1990.607 (1004) ***		30.761 (30)	0.251	0.066	0.897	0.051
Model-β	1997.632 (1005) ***		7.025 (1)	0.008	0.070	0.896	0.051

Notes. Model-0 is the baseline model with no cross-group constraints. Model-λ is the metric invariance model with factor loadings constrained to be equal across groups. Model-β is the structural invariance model with the path coefficient between DLC and IDLE constrained to be equal across groups. *** *p* < 0.001.

## Data Availability

The original contributions presented in this study are included in the article. Further inquiries can be directed to the corresponding author.
